# Successful pulmonary arterial embolization followed by curative surgery for a lepidic predominant lung adenocarcinoma with severe hypoxemia

**DOI:** 10.1186/s12893-018-0351-1

**Published:** 2018-04-10

**Authors:** Louise Sebane, Mostafa El-Hajjam, Philippe Puyo, Elisabeth Longchampt, Etienne Giroux Leprieur

**Affiliations:** 1 Department of Respiratory Diseases and Thoracic Oncology, APHP - Ambroise Pare Hospital, 9 Avenue Charles de Gaulle, 92100 Boulogne-Billancourt, France; 2 Department of Radiology, APHP - Ambroise Pare Hospital, Boulogne-Billancourt, France; 3 Department of Thoracic Surgery, Foch Hospital, Suresnes, France; 4 Department of Pathology, Foch Hospital, Suresnes, France; 5 EA4340, UVSQ, Paris-Saclay University, Boulogne-Billancourt, France

**Keywords:** Thoracic surgery, Non-small cell lung cancer, Lepidic adenocarcinoma, Hypoxemia, Arterial embolization

## Abstract

**Background:**

Lepidic predominant adenocarcinoma is characterized by frequent refractory hypoxemia due to intrapulmonary shunting. Severe hypoxemia can induce perioperative complications in case of thoracic surgery.

**Case presentation:**

We report a case of a 67 year-old woman with localized lepidic adenocarcinoma in the right lower lobe with severe hypoxemia. A selective arterial lung embolization allowed an instantaneous correction of the hypoxemia, and a curative lobectomy was safely performed 1 week after without any complication. The staging was pT3N0M0, and the patient received adjuvant chemotherapy.

**Conclusions:**

This is the first case-report of successful endovascular embolization before curative surgery for a lepidic predominant lung adenocarcinoma.

## Background

Adenocarcinoma is the most frequent histological subtype in non-small cell lung cancer (NSCLC). Lepidic predominant subtype is a particular form of invasive lung adenocarcinoma, developed initially from Club cells and/or type II pneumocytes [1]. The alveolar spreading induces a specific clinical and radiological presentation, with dyspnea, cough, ground-glass attenuations and alveolar consolidations on CT-scan [1]. The presence of severe hypoxemia represents an issue in case of localized tumour with indication of surgical resection. We present here the case of a 67 year-old women with localized lepidic predominant adenocarcinoma and severe hypoxemia due to intrapulmonary shunting, who was successfully treated by pulmonary arterio-embolization followed by curative lower lobe resection.

## Case presentation

**Fig. 1:**
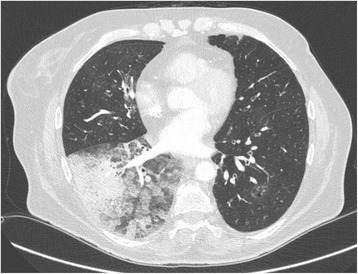
CT-scan showing ground-glass opacities and alveolar consolidation in the right lower lobe

**Fig. 2:**
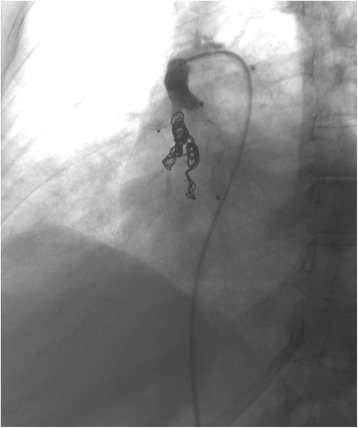
Successful intravascular occlusion of the right lower lung artery by coils and plugs

A 67 year-old woman was admitted to the Department of Respiratory Diseases and Thoracic Oncology for dyspnea, increasing over a few weeks, without infectious context. Her medical history was a breast cancer in 2006 treated by surgery, chemotherapy and radiotherapy, without evidence of recurrence, diabetes, systemic hypertension and an active smoking at 40 packet-years. The oxygen saturation at the admission was at 80% in room air, with need for supplementation of oxygen at 6 l/min. Arterial blood gas on 6 l/min O2 supplementation showed a PO2 of 70 mmHg, a PCO2 of 39 mmHg and pH of 7.42. Functional tests showed a moderate obstructive syndrome, with a forced expiratory volume in 1 sec (FEV1) on vital capacity ratio at 66%, and a FEV1 at 1350 ml (62%). Unfortunately, the evaluation of diffusing capacity of the lung for carbon monoxide (DLCO) was not feasible at the time of the diagnosis, due to the severity of dyspnea. The CT-scan revealed ground-glass opacities and alveolar consolidation in the right lower lobe (Fig. 1). Transthoracic echocardiography demonstrated normal left ventricular ejection fraction. Cytology on bronchial aspiration and right lower lobe transbronchial biopsy found adenocarcinomatous cells with positive staining for *Thyroid Transcription Factor-1* (TTF-1) in immunohistochemistry (IHC). Brain CT-scan was normal, and PET-CT showed localized hypermetabolism on the right lower lobe. A pulmonary angiography was performed. The temporary balloon occlusion of the right lower pulmonary artery showed a rapid increase of the oxygen rate from 90% on oxygen 6 L/mn to 100% in room air, and the patient underwent thereafter an intravascular occlusion of this artery by coils and plugs (Fig. 2). The oxygen saturation increased instantly to 100% at room air; arterial blood gas at room air showed a PO2 of 82 mmHg, a PCO2 of 33 mmHg and a pH of 7.43. One week later, a lower right lobectomy with mediastinal lymphadenectomy was performed without post-operative complication. The pathological study showed a 9 cm-sized lepidic predominant adenocarcinoma (pT3N0M0), with necrotic and hemorrhagic changes due to the embolization, and *Cytokeratin 7* (CK7) and TTF-1 positive IHC staining. The patient received adjuvant cisplatin and vinorelbine chemotherapy.

## Discussion and conclusion

The deep hypoxemia observed occasionally in lepidic lung adenocarcinoma is due to the filling of alveolar spaces by tumor cells, along with normal perfusion of these territories. This phenomenon induces a veno-arterial shunting, with hypoxemia and normocapnia. The first description was made in 1969 by Wolinsky and Williams, with a case of diffuse bronchio-alveolar lung adenocarcinoma showing hypoxemia, normal lung angiogram and normal pulmonary function tests [2]. Since then, only a few cases of lepidic adenocarcinoma treated by surgery have been reported [3–10]. The surgery induced an improvement of hypoxemia and symptoms in all patients, with an overall survival after surgery between 21 days and 24 months in such a palliative context. However, the management of the hypoxemia due to intrapulmonary shunting with perfusion of a non-ventilated lobe, is not codified. The possibility of surgical ligation of the vessels in the lower lobe during the thoracotomy was considered for our patient, but we chose to correct the deep hypoxemia before the surgery, as deep hypoxemia can be a source of complications during the perioperative time, and is reported to be a prognostic factor [11–13].

The temporary balloon occlusion is a good indicator of the potential benefit of surgery or embolization. Wartzki et al. described a case of left pneumonectomy for a stage III adenocarcinoma performed after a temporary balloon occlusion showing a correction of the shunting [8]. One case of palliative embolization was reported, in 2015 [9]. In this case of an extensive bilateral lung disease, the procedure failed to demonstrate a PO2 improvement but it induced a clear improvement of the dyspnea. Finally, the association of the two procedures (embolization then surgery) has only been reported in one case [10]. It was also a palliative situation and the association of these two procedures allowed an improvement of the dyspnea and the PO2.

Our case is the first to describe the successful pre-surgical management of a lepidic predominant adenocarcinoma with a curative objective. The occlusion of the right lower lung artery allowed in our patient a rapid and prolonged improvement of the haematosis with a complete correction of the hypoxemia. The surgery was then performed safely with optimal respiratory conditions and without any complication.

In conclusion, the occlusion of the right to left shunt by endovascular embolization is a safe and efficient procedure before curative surgery for lepidic predominant lung adenocarcinoma.

## Abbreviations

CK7: Cytokeratin 7
CT-scan: Computerized-tomography scan
DLCO: Diffusing capacity of the lung for carbon monoxide
FEV1: Forced expiratory volume in one second
IHC: Immunohistochemistry
NSCLC: Non-small cell lung cancer
TTF1: Thyroid Transcription Factor-1
